# Urothelial Bladder Cancer With Cardiac Metastasis: A Case Report

**DOI:** 10.7759/cureus.103414

**Published:** 2026-02-11

**Authors:** Oualid Bounouar, Anouar El Moudane, Elie Andrea, Ahmed Jdaini, Ali Barki

**Affiliations:** 1 Urology, Centre Hospitalier Universitaire Mohammed VI Oujda, Oujda, MAR; 2 Urology, Mohammed VI University Hospital, Oujda, MAR; 3 Urology, Centre Hospitalier de Compiègne, Compiegne, FRA; 4 Urology, Mohammed VI University Medical Center, Oujda, MAR; 5 Urology, Faculty of Medicine and Pharmacy, Mohammed the First University, Oujda, MAR

**Keywords:** 18-fdg pet scan, cardiac metastasis, case report, hematuria, urothelial carcinoma

## Abstract

We report a case with a large muscle-invasive bladder TCC and cardiac and disseminated metastases. A 74-year-old man was referred to our department due to a decline in general health, associated with macroscopic hematuria for the past two months, in the context of urinary infection. CT imaging revealed a large bladder tumor with bilateral hydronephrosis, suspicion of peritoneal carcinomatosis, and locoregional lymphadenopathies suggesting secondary involvement. Histology of the bladder tumor confirmed the diagnosis of high-grade infiltrating urothelial carcinoma. The 18-FDG PET scan detected diffuse lymph node, hepatic, and bone metastases, as well as peritoneal carcinomatosis and metastases in the left ventricle and atrium. Chemotherapy was planned for our patient; unfortunately, the patient passed away rapidly due to renal and cardiac complications before treatment could begin.

## Introduction

Metastasis of urothelial carcinoma of the heart is an uncommon yet clinically significant complication. Most cardiac metastases remain asymptomatic until advanced stages, and such cases are often associated with a poor prognosis. Although any malignant neoplasm has the potential to spread to the heart, cardiac metastases most frequently arise from melanoma, carcinomas (particularly of the lung, breast, and esophagus), and, less commonly, colorectal as well as hematologic malignancies such as leukemia and lymphoma [1,2], while urothelial carcinoma is a rare cause of cardiac metastasis. Therefore, it is essential to identify subclinical cardiac metastases, and timely initiation of systemic antineoplastic therapy is essential to improve overall patient outcomes.

## Case presentation

A 74-year-old male was referred to our department for gross hematuria for two months ago associated with a history of recent urinary tract infections. With no previous medical history except a heavy smoking history of 40 packs a year, quit 20 years ago, and 10 kg weight loss over the last few months has also been reported by the family. He described some urinary frequency and urgency for one year, for which he did not seek medical care. Physical examination showed an OMS 3 patient with severe dehydration and diffuse abdominal tenderness without contracture. Digital rectal examination showed a small, unsuspicious prostate with severe pelvic floor induration. No peripheral palpable lymph nodes.

**Figure 1:**
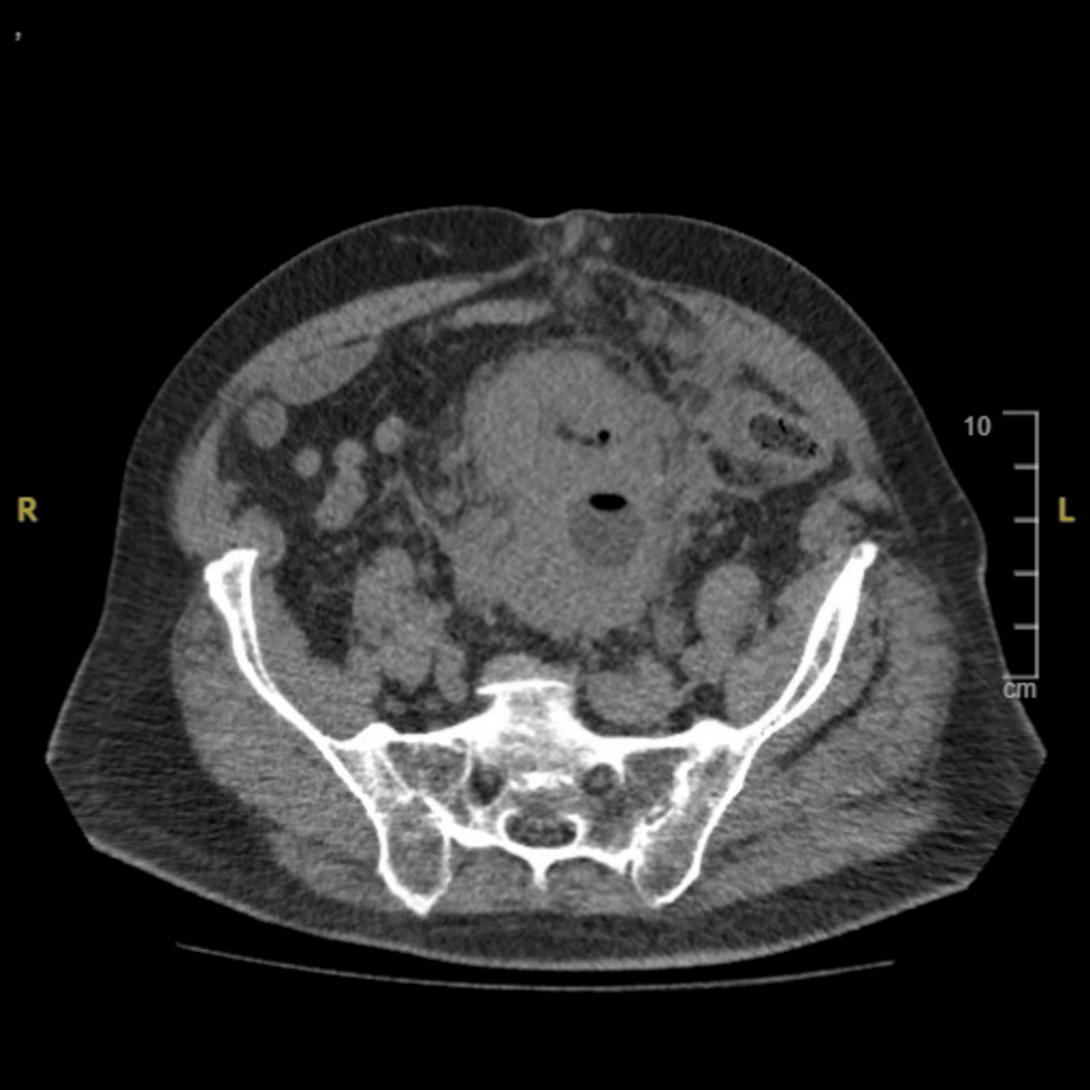
Axial view of abdominopelvic computed tomography showing a large bladder tumor.

**Figure 2:**
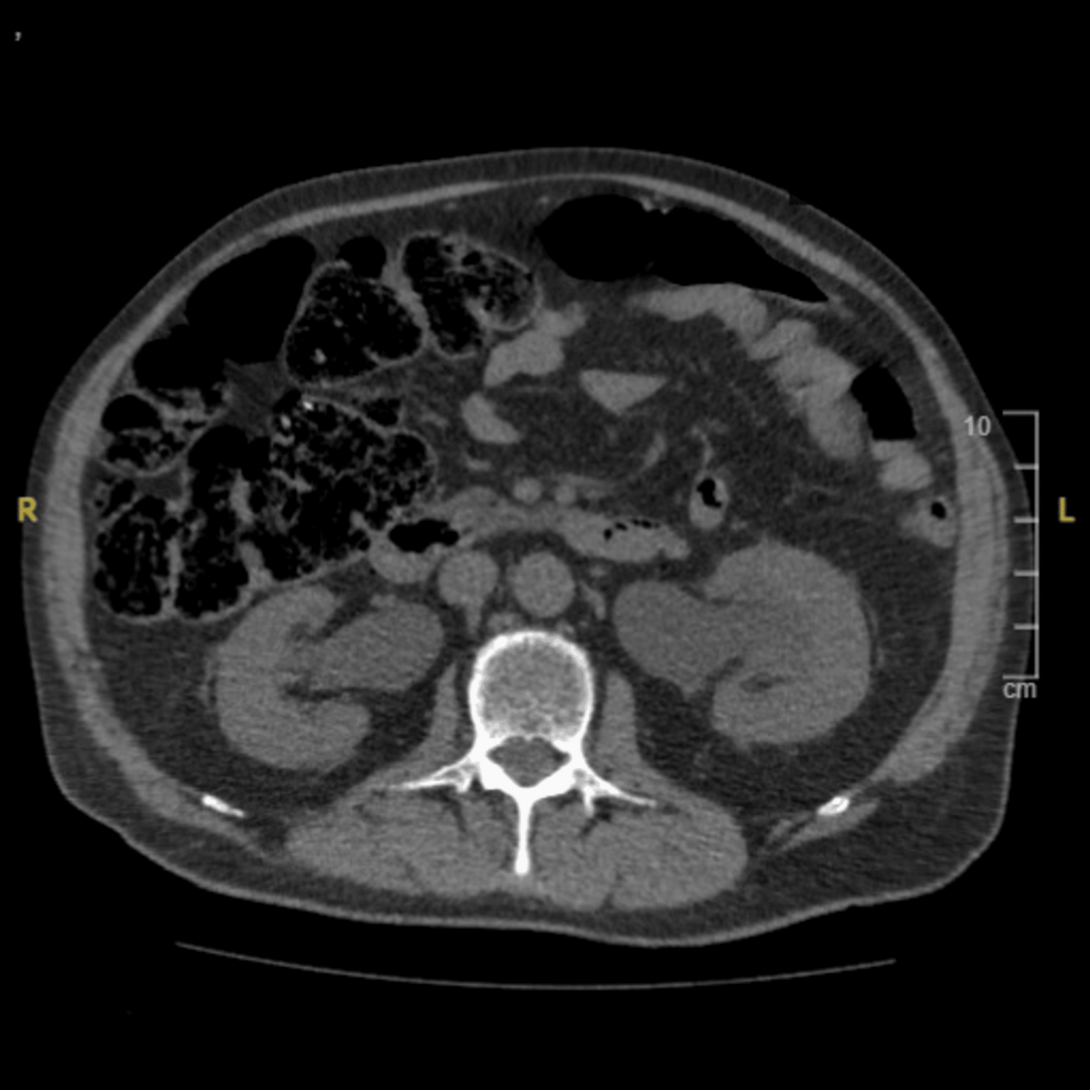
Axial view of abdominopelvic computed tomography showing a bilateral hydronephrosis.

A blood test showed moderate renal insufficiency (GFR 40 ml/min) with anemia (hemoglobin 7 g/dL). An abdominal and pelvic CT scan showed a huge bladder tumor with bilateral hydronephrosis, bilateral pelvic metastatic lymph nodes, and peritoneal carcinosis (Figures 1, 2).

**Figure 3:**
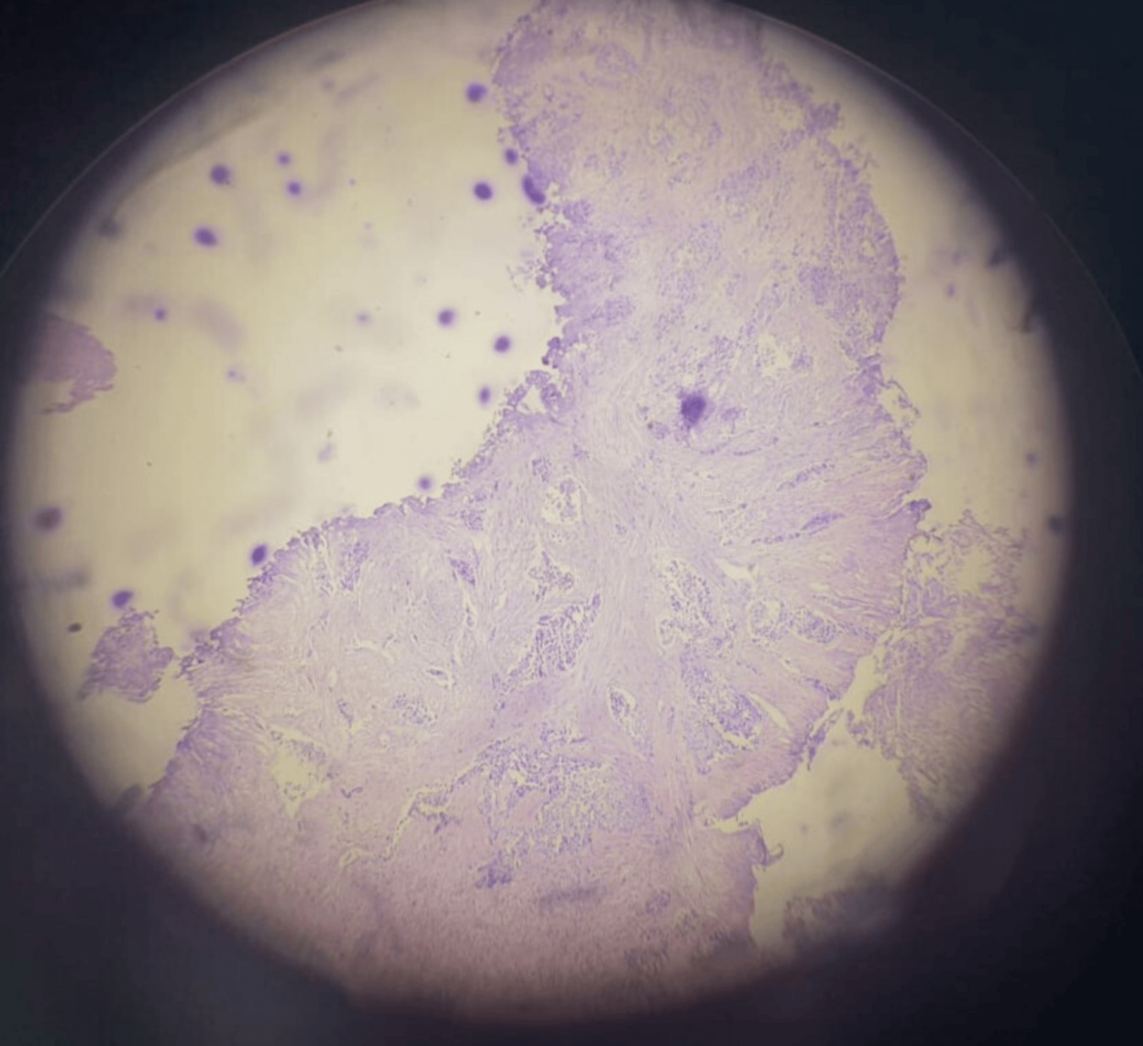
Histological section showing a high-grade urothelial carcinoma with muscle infiltration.

**Figure 4:**
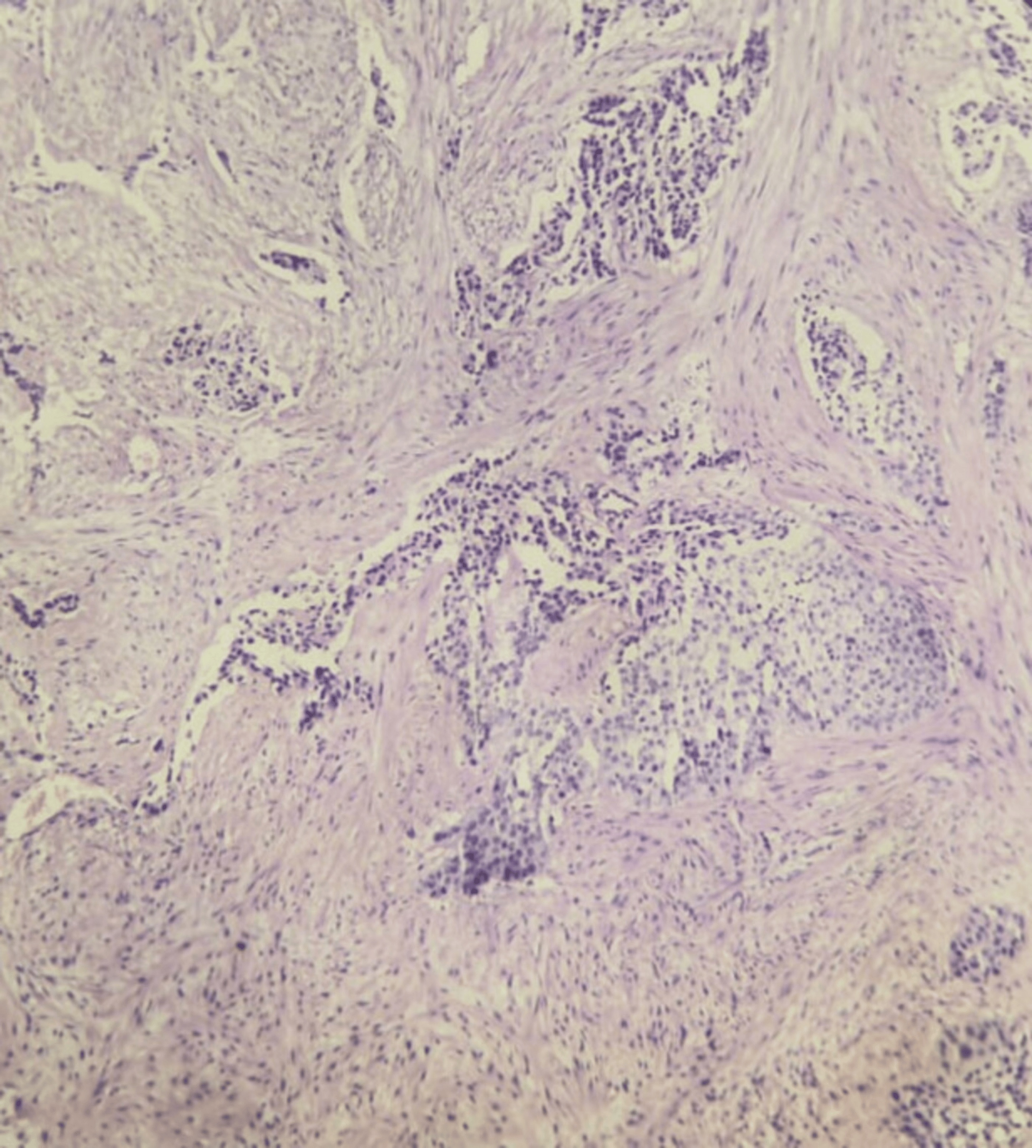
Histological section showing urothelial carcinoma with the presence of atypical cells.

We underwent a transurethral resection of a bladder tumor, showing a large nodular and papillary tumor occupying all the bladder walls, without any normal mucosa. Ureteral orifices were invaded and couldn’t be identified despite deep resection and injection of indigo, carmine, and blue. Percutaneous nephrostomy was refused by the patient with initially moderate renal failure and a GFR of 40 ml/min. Pathological examination confirmed a high-grade, muscle-invasive urothelial carcinoma of the bladder, classified as pT2 (Figures 3, 4).

**Figure 5:**
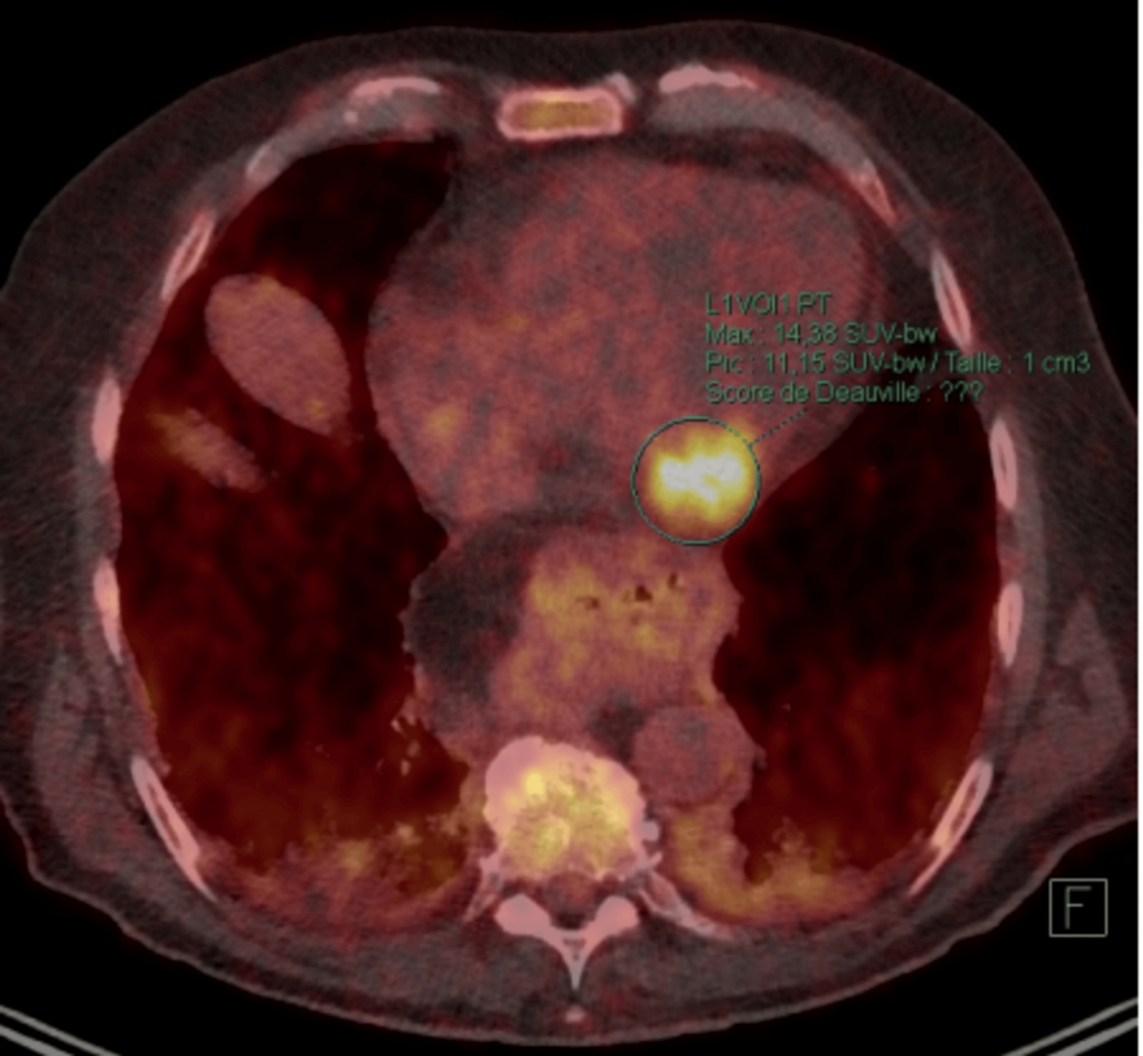
An 18FDG PET scan showing a hypermetabolism area in the left cardiac ventricle in axial view.

**Figure 6:**
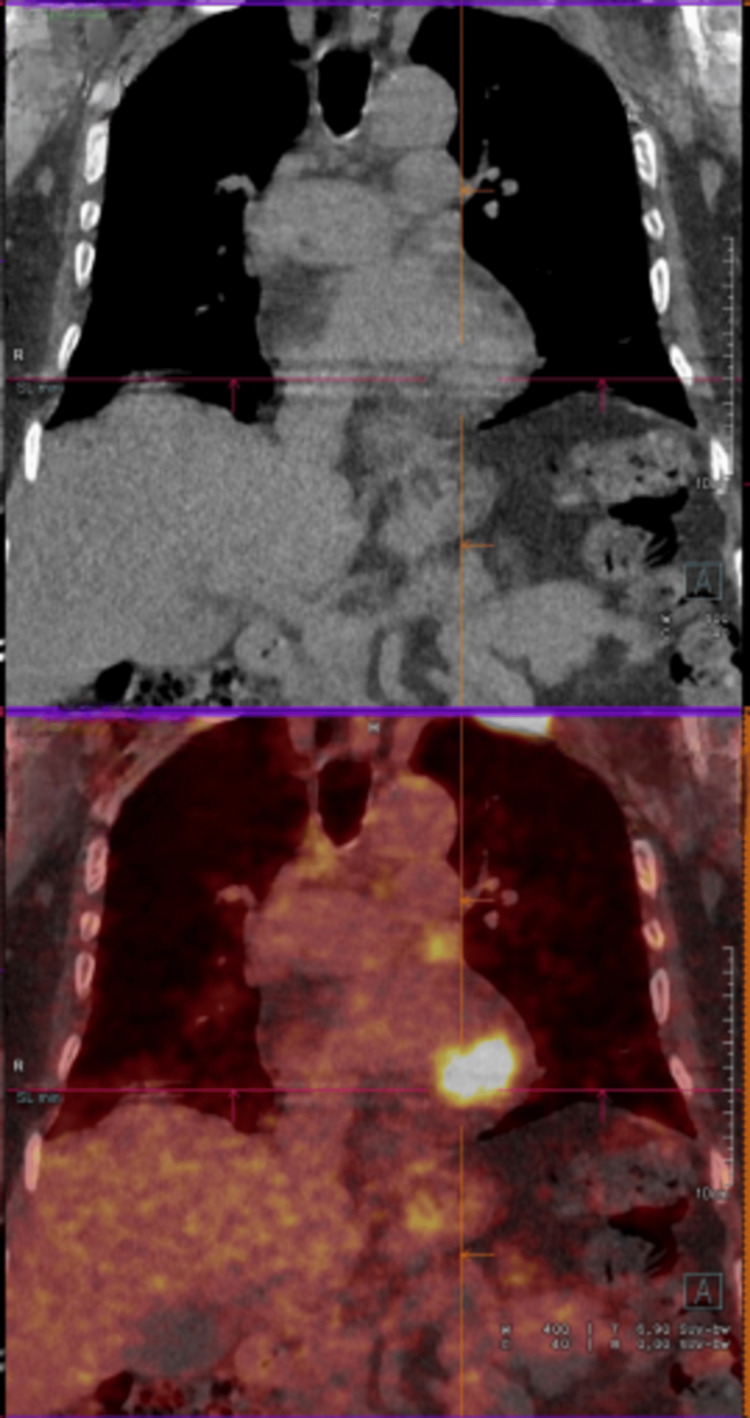
PET-FDG with an area of hypermetabolism in the left ventricle in the coronal section.

An 18-fluoro-deoxyglucose positron emission tomography (18FDG PET scan) was subsequently requested, showing widespread metastases in the left ventricle, left atrium, bilateral pelvic lymph nodes, liver, and bones (Figures 5, 6).

A cisplatin-based chemotherapy regimen was decided on with the oncology team after percutaneous nephrostomy to optimize renal function, but the patient's severely altered conditions led to rapid death from cardiac and renal complications.

## Discussion

Bladder cancer, also referred to as urinary bladder cancer, ranks as the 9th most common cancer globally, with its incidence steadily increasing, particularly in developed countries. In the United States, bladder cancer ranks as the sixth most frequently diagnosed malignancy, accounting for approximately 3% of all new cancer diagnoses. Urothelial carcinoma comprises the vast majority of cases, while squamous cell carcinoma is more prevalent in regions where schistosomiasis infection is endemic [3,4].

A study published in 2023 analyzed 30 patients with cardiac metastases from transitional cell carcinoma (TCC) reported between 1934 and 2023, highlighting the rarity of this condition. Most patients were men (77.8%) with a mean age of 65.5 years, and over half of the cases (57.9%) were diagnosed post-mortem at autopsy [5]. Cardiac metastases often remain subclinical, with many cases identified only through autopsy. However, they can present with a wide range of clinical manifestations contingent upon the tumor's size, anatomical site, and the severity of myocardial or pericardial infiltration. In some instances, these lesions are detected incidentally during diagnostic staging or longitudinal surveillance of the primary malignancy [6].

In a large autopsy-based analysis published in 2007, Bussani et al. evaluated 18,751 in-hospital deaths and identified malignant neoplasms in 7,289 cases. Cardiac metastases were observed in 662 patients, representing 9.1% of individuals with malignancy. Lung cancer was the most frequent primary tumor associated with cardiac metastases (39.2%), followed by breast cancer (10.0%), mesothelioma (9.4%), and lymphoma or leukemia (10.0%). The authors further demonstrated that mesothelioma, melanoma, and lung cancer show a higher tendency to metastasize to the heart. In contrast, cardiac metastases originating from urothelial carcinoma were uncommon, occurring in only 12 of 662 cases (1.8%), underscoring the rarity of urothelial carcinoma as a source of cardiac metastasis [1]. Pericardial involvement is the most commonly reported site of cardiac metastasis, followed by the myocardium; conversely, the endocardium is the least frequently affected location [7].

Patients presenting with cardiac metastases are frequently asymptomatic, with detection primarily occurring during post-mortem examinations, as previously discussed. In cases where symptoms are present, they tend to be nonspecific and often overlap with those of metastases in other organs, which may already be prevalent by the time cardiac involvement occurs. This can account for the frequent underdiagnosis of cardiac metastases. However, when metastases are localized to the heart, specific symptoms may arise depending on the anatomical site of the metastatic lesions. For instance, pericardial metastasis may manifest similarly to pericarditis, potentially leading to pericardial effusion and, in severe cases, tamponade [8]. Involvement of the myocardium may induce arrhythmogenic events, including life-threatening conditions such as ventricular fibrillation or complete atrioventricular block [9]. Additionally, myocardial infiltration can compromise cardiac output, presenting as congestive heart failure. Endocardial metastases, on the other hand, can result in obstructed left or right ventricular outflow, leading to cardiogenic shock [10]. Any of these symptoms, when observed in a patient with a known malignancy, should prompt consideration of cardiac metastasis.

Due to the advanced stage at diagnosis, curative surgery is feasible for only a small number of patients with cardiac metastatic tumors. This is particularly true when the disease is confined to the heart and the primary tumor is under control, or when metastases cause obstruction in the right ventricular outflow tract. In such cases, surgical debulking can alleviate symptoms and extend survival, a benefit also offered by chemotherapy, which our patient chose [11]. In general, surgical interventions primarily focus on relieving recurrent pericardial effusions or tamponade, typically performed via subxiphoid pericardiotomy.

## Conclusions

Cardiac metastasis from transitional cell carcinoma (TCC) is a rare occurrence, with most patients being diagnosed at advanced stages and often presenting with severe clinical symptoms. In most cases, overall survival is limited to a short duration, typically ranging from several weeks to a few months. Echocardiographic evaluation, including transthoracic and transesophageal modalities, may demonstrate myocardial metastatic masses, especially in relation to clinical signs of infection, which can mimic endocarditis or myocarditis. In general, establishing a clinical diagnosis of metastasis of the heart is often problematic, as these lesions typically remain clinically silent in the early stages. For patients with cardiac metastases, treatment options are primarily limited to chemotherapy and palliative care for symptom management. In certain cases, surgery may be considered to alleviate symptoms.
